# Correction: Fluorido-bridged robust metal–organic frameworks for efficient C_2_H_2_/CO_2_ separation under moist conditions

**DOI:** 10.1039/d3sc90025h

**Published:** 2023-02-08

**Authors:** Yi-Ming Gu, You-You Yuan, Cailing Chen, Sheng-Sheng Zhao, Tian-Jun Sun, Yu Han, Xiao-Wei Liu, Zhiping Lai, Shu-Dong Wang

**Affiliations:** a Dalian National Laboratory for Clean Energy, Dalian Institute of Chemical Physics, Chinese Academy of Sciences Dalian 116023 China wangsd@dicp.ac.cn; b University of Chinese Academy of Sciences Beijing 100049 China; c Core Laboratory, King Abdullah University of Science and Technology (KAUST) Thuwal 23955-6900 Saudi Arabia; d Advanced Membranes and Porous Materials Center, Division of Physical Sciences and Engineering, King Abdullah University of Science and Technology (KAUST) Thuwal 23955-6900 Saudi Arabia zhiping.lai@kaust.edu.sa xiaowei.liu@kaust.edu.sa

## Abstract

Correction for ‘Fluorido-bridged robust metal–organic frameworks for efficient C_2_H_2_/CO_2_ separation under moist conditions’ by Yi-Ming Gu *et al.*, *Chem. Sci.*, 2023, https://doi.org/10.1039/d2sc06699h.

The name of the third author, Cailing Chen, was spelled incorrectly in the original version of this manuscript. This correction notes that the author is Cailing Chen and replaces the original authorship list.

Within the Results and discussion section of the manuscript, the sentence “As shown in Fig. 1d and e, the ordered structure of DNF-9(Fe) was observed by high resolution transmission electron microscopy (HRTEM)” is incorrect as the MOF studied is DNL-9(Fe). The corrected sentence is as follows:

“As shown in Fig. 1d and e, the ordered structure of DNL-9(Fe) was observed by high resolution transmission electron microscopy (HRTEM)”.

The Royal Society of Chemistry apologises for these errors and any consequent inconvenience to authors and readers.

## Supplementary Material

